# The possible application of virtual reality for managing anxiety in athletes

**DOI:** 10.3389/fspor.2025.1493544

**Published:** 2025-02-20

**Authors:** Melinda Trpkovici, Alexandra Makai, Viktória Prémusz, Pongrác Ács

**Affiliations:** ^1^ Doctoral School of Health Sciences, Faculty of Health Sciences, University of Pécs, Pécs, Hungary; ^2^ Faculty of Health Sciences, Institute of Physiotherapy and Sport Science, University of Pécs, Pécs, Hungary; ^3^ Physical Activity Research Group, Szentágothai Research Centre, Pécs, Hungary

**Keywords:** athlete anxiety, sports psychology, self-confidence, concentration, virtual reality

## Abstract

**Introduction:**

One of the most effective techniques is “stress inoculation” training (SIT), which is increasingly utilized to reduce anxiety and enhance athletic performance. The aim of our research was to investigate the extent to which the stress situation we created in virtual reality evokes psychological responses in athletes, compared to the responses they experience during a competitive match.

**Methods:**

The sample consisted of 24 female athletes with an average age of 18.71 ± 5.42 years. Of these, 9 were elite basketball players, 8 were table tennis players, and 7 were handball players. All participants completed the Athlete Anxiety Questionnaire, designed to measure anxiety in high-stakes situations and assess levels of concentration and self-confidence during matches. Additionally, within the virtual reality environment we created, athletes were exposed to stress-inducing factors scientifically proven to elicit stress responses. Paired-sample *t*-tests were used to examine differences between measurements (match and virtual reality scenario), and ANOVA tests were used to compare differences between athletes groups (basketball players, table tennis players and handball players).

**Results:**

Our findings indicated that the sports stress scenario simulated in virtual reality triggers stress responses in athletes comparable to those experienced during actual competitive matches. No significant difference was detected in any factor between the total scores of the tests completed after the virtual reality session and those completed after the match (*p* > 0.05). The greatest impact of VR on cognitive anxiety was observed regarding the fear of mental block or choking during a match, received an average score higher than that of the match (1.75 ± 1.032 vs. 1.38 ± 0.711 respectively, *p* = 0.04)

**Conclusion:**

Based on these results, we can conclude that the sports stress scenario generated by virtual reality can indeed induce a comparable level of stress in athletes compared to actual matches. Therefore, virtual reality technology shows promise as a tool for enhancing athletes' stress management skills and could be a significant asset in sports psychology preparation processes.

## 1 Introduction

Exercise generally positively affects health, including physical and mental well-being (1); however, the specific circumstances of elite sports can sometimes lead to anxiety. “Elite sports” typically refer to professional-level competition, which involves high expectations and performance pressures that can induce stress and anxiety among participants (2, 3). Anxiety is a widely studied variable in sports psychology and has been the focus of numerous research studies (2, 4–7). Several reviews have been published to offer an insightful and informative perspective on the correlation between competitive anxiety and performance (4, 5, 8–13). Anxiety is a distressing, tense emotional condition characterized by heightened activation of the autonomic nervous system, coupled with negative feelings and thoughts (14, 15). Athletes frequently encounter performance-related concerns that result in increased anxiety, characterized by muscle tension, sweaty palms, and pessimistic thoughts regarding critical moments and their own capabilities (16, 17). Spielberger has distinguished between two types of anxiety: trait anxiety and state anxiety (18). State anxiety manifests itself only in specific situations (e.g., competitions), while trait anxiety reflects an individual's general tendency towards anxiety, often considered a personality trait. These different types of anxiety have long been recognized. Additionally, distinctions can be made between cognitive anxiety and somatic anxiety (8, 11, 19–23). Cognitive anxiety presents as persistent worry, rumination, and fixation on something, leading to decreased concentration, impaired performance, and an increased likelihood of making mistakes (24, 25). In contrast, somatic anxiety involves the physical manifestations of anxiety, primarily linked to the direct activation of the autonomic nervous system (23, 26). Gould et al. (27) believed that somatic anxiety has a direct impact on performance (27). Although mild competition anxiety can be beneficial for athletes as it aids concentration, higher levels of anxiety reduce concentration abilities, leading to errors and decreased performance (28).

Sports psychologists have suggested various stress management techniques over the years, but one of the most successful is “stress inoculation training” (SIT). Developed in the late 1970s by Donald Meichenbaum, a psychologist at the University of Waterloo in Canada. The stress inoculation training is based on the fact that if individuals are exposed to manageable but gradually increasing amounts of stress, their immunity to stress is strengthened (29). The key to stress inoculation, however, is not just that the level of stress is gradually increased during training, but also that in this environment, athletes learn to manage psychological stress and develop skills aimed at enhancing performance. These skills include the use of productive thoughts and coping strategies. The general stress inoculation process typically consists of three stages: the first is the Conceptualization phase. This initial stage involves educating individuals about the nature and effects of stress. Therapists use cognitive interviews to help patients identify their specific stress triggers and understand their stress responses. The aim is to thoroughly understand how stress impacts them and prepare them for learning coping techniques. The second is the Skills Acquisition phase. During this stage, the athlete learns to use coping skills. These include relaxation techniques, cognitive restructuring to challenge and change negative thoughts, deep breathing exercises, muscle relaxation, and positive self-talk. Techniques like role-playing and stress simulation are used to practice these skills in a controlled setting. The third is the Application phase. In the final phase, individuals apply their newly learned skills in real-life scenarios. This includes role-playing, visualization, and *in vivo* exposure to actual stressors. The focus is ensuring that individuals can effectively use their coping strategies outside therapy sessions. This phase also incorporates relapse prevention, helping individuals prepare for potential setbacks and reinforcing their coping techniques. Here, the athlete practices these skills, initially in low-stress situations and then in situations where the stress level gradually increases (29). This method proves effective in numerous scientific fields, e.g., clinical (30) and sport (31). The significant advantage of stress inoculation training is its high flexibility and wide applicability. This method can be used to address various stress-related issues such as pain reduction (32) and overcoming fear (31). Due to this flexibility, stress inoculation training can be particularly valuable in sports, where athletes often experience performance anxiety due to fears such as fear of injury, pain, or failure. Practical findings showed that stress inoculation training effectively reduces performance anxiety and state anxiety while enhancing performance. This method helps individuals manage the stress encountered in competitive situations (33, 34). Building on the success of SIT, during training sessions, athletes are exposed to various stressors to enhance their coping abilities against stress, preparing them to manage effectively the high-pressure situations caused by competition. In the research of Reeves et al. (35), sports psychologists applied the SIT program to a football team. During the training, the athletes were initially exposed to milder stressful situations, such as having to perform certain technical tasks under time pressure. Later, more complex and intense stressors were introduced, such as performing in front of an audience or dealing with provocative behavior from competitors. Before each session, the athletes were given a situational exercise to help them learn how to respond to stress and use coping techniques.

The results of the research showed that the athletes participating in the SIT program made greater progress in stress management and were less affected by the high pressure experienced in competition situations. Techniques learned during training, such as relaxation, positive self-talk, and mental visualization, helped the athletes manage the tension associated with competitive situations and improved their performance in important matches.

However athletes cannot be exposed to competitive or stressful situations on a daily basis, making it worthwhile to utilize the advancements of Virtual Realitiy (VR) for stress management. A modern and innovative research area is the study of the effects of virtual reality (VR). Virtual reality was first used in sports research in the 1990s, but it has recently gained significant interest again. VR refers to a computer-simulated environment that aims to create a sense of being mentally or physically present in another place (36, 37). In the early 2010s, researchers already began investigating the impact of VR on sports performance, although these studies were mostly anecdotal in nature (38, 39). VR provides the opportunity to simulate and train responses to stressful situations in a controlled environment. This can help individuals strengthen their stress tolerance and develop stress management strategies. Previous research findings have also supported that virtual sports environments elicit measurable stress responses from athletes. In Stinson and Bowman's research (40), they discovered that a 3D realistic sports environment triggered measurable anxiety responses in athletes at both physiological and psychological levels. This finding suggests that such a virtual setting can effectively simulate the pressures and challenges of real-life sport scenarios. The physiological responses included increased heart rate and elevated cortisol levels, while the psychological responses were characterized by heightened feelings of anxiety and stress. These results underscore the potential of using advanced virtual reality environments as tools for training and preparing athletes for competitive situations (40).

Virtual reality can be widely applied in various interventions, with numerous studies demonstrating its effectiveness. Harrison et al. (41) examined how a VR relaxation intervention influences perceived anxiety levels. They utilized the Mental Rediness Form-3 (MRF-3) as a measurement tool, which assesses cognitive anxiety, somatic anxiety, and self-confidence (42). The results demonstrated that the VR intervention significantly lowered both cognitive and somatic anxiety while boosting self-confidence. Moreover, all participants reported that VR helped them relax (41). Another study assessed the effects of a training program using virtual reality simulation on competitive anxiety. The sample consisted of 10 table tennis players aged 13–15 years. Researchers utilized virtual reality in the training program over a period of 6 weeks, with sessions conducted 5 times per week. Performance anxiety was measured using the Competitive State Anxiety Inventory-2 (CSAI-2) questionnaire. Results indicated that the program incorporating virtual reality simulation had a positive impact on reducing performance anxiety. Based on these findings, the researchers recommended the use of modern technological tools in sport psychology (43). In 3D technology, therefore, there is an opportunity for stress training (44–46). SIT is VR can also be used to practice specific skills. Cycling and running have been the most commonly used sports tasks so far, but rowing, weightlifting, and golf have also been examined. These sports include elements of endurance and perseverance, making them relatively easy to translate into a virtual environment. The load surface of a treadmill or ergometer can easily monitor information such as speed and other performance metrics (e.g., cadence), which can then be translated into virtual movements (47). The presence of others in the virtual environment is also an essential feature of the VR environment. In fact, the presence of others may be even more important than the capability of the VR system itself to induce feelings of immersion or presence (48). The presence of others in the virtual environment can directly exert pressure on competitive performance (49).

Our research builds upon the theoretical considerations and research findings outlined in the introduction. In our research, we have set three goals:

(a) Examine and evaluate the psychological responses triggered by a stress situation created in virtual reality (VR sports stress scenario) in athletes.
(b) Examine whether there is a significant difference between those who work with a sports psychologist and those who do not.
(c) Compare athletes from the three sports (basketball, table tennis, handball) in terms of their anxiety scores.

## 2 Methods

### 2.1 Study design and participants

Planning the examination and reporting the results were based on the STROBE checklist guidelines (50). The cross-sectional quantitative study was conducted between December 2019–December 2020 in Pécs, Hungary. Ten participants were excluded because of inadequately filled questionnaires. The participants did not answer several questions, rendering their questionnaire incomprehensible. The final sample consisted of 24 female athletes with an average age of 18.71 (±5.42) years. Of these, 9 were elite basketball players, 8 were table tennis players, and 7 were handball players. All participants completed the Athlete Anxiety Questionnaire, designed to measure anxiety in high-stakes situations and assess levels of concentration and self-confidence during matches. Inclusion criteria.

To qualify for participation, individuals needed to meet specific criteria. They had to be certified athletes actively involved in a sports club for at least one year, attending training sessions at least three times a week, each lasting one and a half hours. Additionally, participants had to be at least 12 years old and were required to sign an informed consent form. For participants under 18 years, parental or guardian written informed consent was mandatory following a detailed study briefing. Exclusion criteria.

Receiving psychiatric treatment and taking medications were identified as exclusion criteria. However, no athletes were excluded based on these criteria.

### 2.2 Procedure of the examination

First, the athletes completed the Athlete Anxiety Questionnaire (AAQ) after the competition, then within two weeks, and also after the VR scenario, they filled out the AAQ again. This questionnaire is suitable for measuring anxiety that appears in high-pressure situations and determining the levels of concentration and self-confidence experienced during the competitive match. The questionnaire contains 20 items, which are organized into four factors and measure: (1) the athlete's cognitive anxiety in high-pressure situations, (2) somatic anxiety, (3) self-confidence, and (4) concentration. According to the instructions, the questionnaire inquires about the feelings, emotions, and thoughts caused by the experienced stress effects. The athlete must indicate on a Likert scale ranging from 1 to 4 how much the given statement applies to them (1 = Not at all; 2 = Somewhat; 3 = Quite a bit; 4 = Very much). For somatic anxiety, the achievable scores range from 3 to 12, for cognitive anxiety from 6 to 24, for self-confidence from 6 to 24, and concentration from 5 to 20. The higher the score the athlete achieves in the factors, the more the given statement applies to them (51). As the total score of the factor measuring somatic anxiety is much lower because it contains fewer questions, we have transposed the results to the AAQ100 in addition to the raw scores, which makes it easier to compare the AAQ scales.

**Figure 1:**
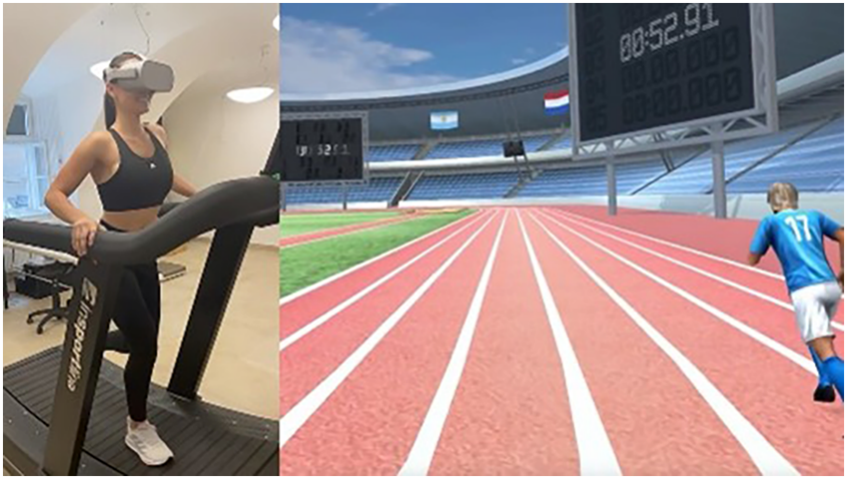
Virtual reality used in the study.

Additionally, within the virtual reality environment we created, athletes were exposed to stress-inducing factors scientifically proven to elicit stress responses. The VR scenario includes stress factors that are scientifically proven (52) to be everyday stressors among athletes (time pressure, unexpected situations, presence of an audience, close score with the opponent, presence of media). A crucial factor is that the athlete is not merely a passive participant in the events but can influence the course of the sporting situation with their current performance. The task is performed by running on a treadmill, and the faster the athlete runs, the quicker they advance in the virtual reality, thereby impacting the events in the virtual environment. Running on the treadmill alongside virtual reality gives participants the opportunity to experience real physical load. This can be particularly important for athletes, as the connection between physical condition and competitive situations plays a key role during actual competitions. We tried to choose a type of movement that appears in most sports, so our method can be used to examine athletes from multiple sports. The virtual sports environment we created is illustrated in the following figure (Figure 1).

The scenario begins with a habituation phase, where the participant can get accustomed to the VR environment. This part is essential, as the new environment itself can be stressful. When the athlete indicates they are “ready”, the race starts with the sound of a starting pistol, and with several opponents competing. After thirty seconds, a stumble occurs, disrupting the athlete's flow. During this time, the tension-building murmur of the audience can be heard, providing negative feedback. Meanwhile, the athlete sees their opponents overtaking them, with some running closely alongside. Throughout the task, the media is present, with a camera moving alongside the runner.

### 2.3 Statistical analysis

Based on the results of the Shapiro–Wilk normality test, paired-sample *t*-tests were used to examine differences between measurements, independent-sample *t*-tests were used to compare differences between those who work with a sports psychologist and those who do not, and ANOVA tests were used to compare differences between athletes groups (basketball players, table tennis players and handball players). Results were considered significant at *p* < 0.05, and data analysis was conducted using IBM SPSS 28.0 software.

### 2.4 Ethical considerations

The study was approved by the Scientific and Research Ethics Committee (Ethical approval number: 8821-5/2019/EÜIG). Prior to the research, participants were informed about the procedure both in writing and verbally, after which they voluntarily provided written consent. For athletes under the age of 18, in addition to the athlete's consent form, a parental consent form was completed, indicating that the parents accepted and agreed to their child's participation in the study. The study adheres to the fundamental principles outlined in the Declaration of Helsinki (53).

## 3 Results

The sample consisted of 24 female athletes, with a mean age of 18.71 (±5.42) years. Of these, 9 were elite basketball players (37.5%), 8 were table tennis players (33.3%), and 7 were handball players (29.2%). Athletes had been practising the sport for an average of 10.46 (±5.12) years. 70.83% of the sample has worked with a sports psychologist before, and 25.0% are currently working with sports psychologist.

Our results showed that the sports stress situation created in virtual reality elicits the same level of stress responses from the athletes as those experienced by the athletes in a competitive match. No significant difference can be detected in any factor between the total scores of the test completed after VR and the test completed after the match (*p* > 0.05) (Table 1).

**Table 1:** Scores achieved on the athletes anxiety questionnaire.

*N* = 24	AAQ scores	Mean	SD	*p*
Somatic anxiety	Match	4.63	1.88	0.219
	VR	3.96	1.92	
Cognitive anxiety	Match	12.63	4.35	0.517
	VR	12.00	4.65	
Self-confidence	Match	16.88	3.18	0.337
	VR	16.21	2.64	
Concentration	Match	15.54	2.23	0.454
	VR	16.00	2.25	

SD, standard deviation.

In the somatic anxiety factor, the highest point was given for question 17 (“*I experienced trembling during the match/competition*”.) for both the match (mean: 1.63 ± 1.01) and VR scenario (mean: 1.50 ± 0.83). This question also showed the smallest difference in scores (0.13 points), indicating that the stress-inducing effect of VR was most comparable to the experiences of the match in this case.

**Figure 2:**
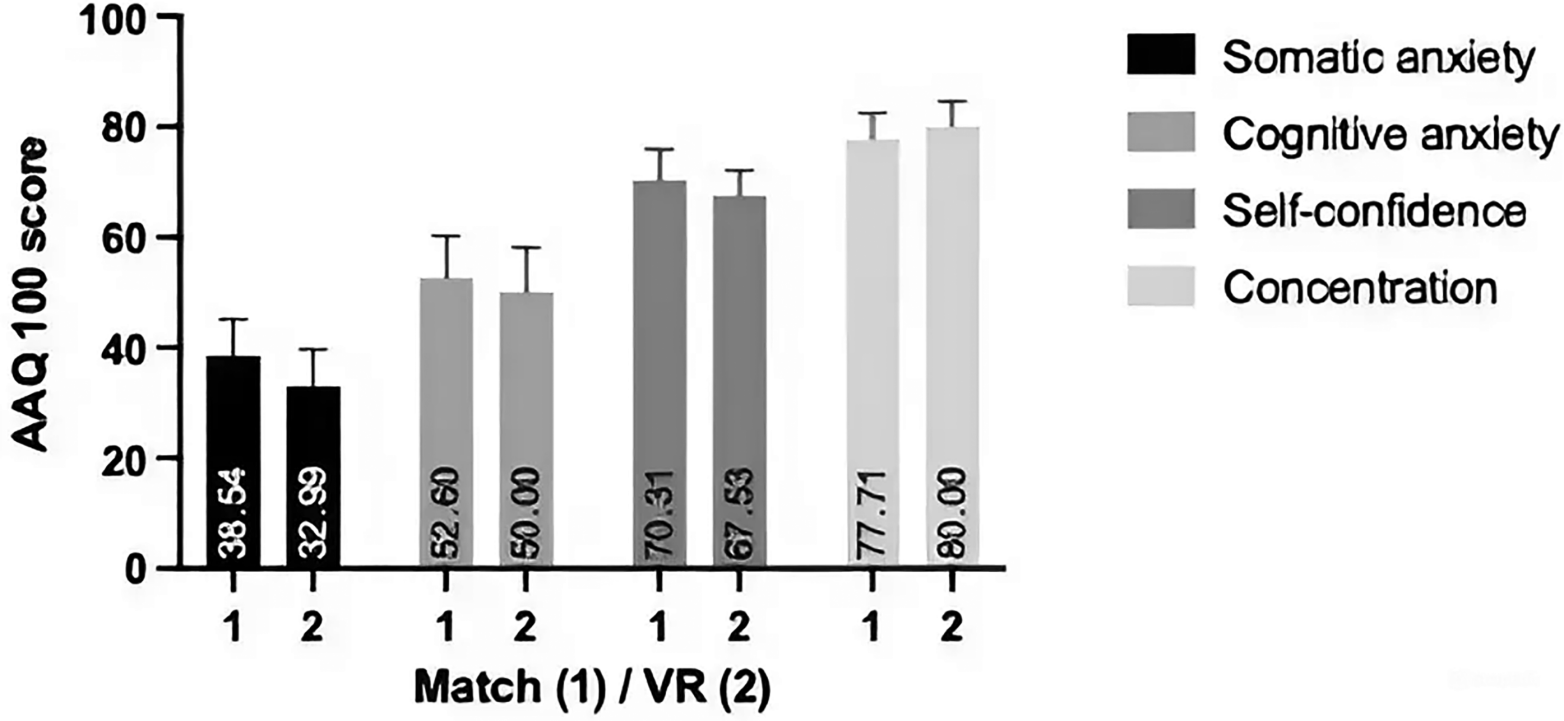
Difference between the VR's and the match's scores in any of the factors of the athlete anxiety questionnaire scores.

In the cognitive anxiety factor, the highest score for the match was given for question 1 (“*I was worried during the match/competition that I wouldn't perform as well as I wanted to*”.) (mean: 2.54 ± 0.779). For VR, the highest scores were given for questions 11 (“*I was afraid during the match/competition that I would perform poorly*”.) and 14 (“*I was afraid during the match/competition that my performance would be disappointing*”.) (mean: 2.17 ± 1.007). The smallest difference between the match and VR scores was for questions 14 (“*I was afraid during the match/competition that my performance would be disappointing*”.) and 18 (“*I was afraid during the match/competition that I would lose*”.) (0.04 points). However, the greatest impact of VR on cognitive anxiety was observed in question 9 (“*I was afraid during the match/competition that I would choke*”). This question reports the fear of mental block or choking during a match because of cognitive anxiety, and received an average score higher than that of the match (match mean: 1.38 ± 0.711; VR mean: 1.75 ± 1.032). In the anxiety measuring factors, only for this question were the scores higher for VR compared to the match (0.37 points) (Figure 2).

We examined whether there was a significant difference between those who work with a sports psychologist and those who do not. Additionally, we compared athletes from the three sports in terms of their anxiety scores. There was no significant difference in any factor between those who work with a sports psychologist, compared to those who do not (*p* > 0.05).

Comparing the three sports, we also found no significant differences between the groups on the anxiety of match (*p* > 0.05). However, table tennis players scored higher on average in the factor measuring cognitive anxiety experienced during the match (match mean: 14.50 ± 2.726) compared to basketball players (match mean: 10.44 ± 4.447) and handball players (match mean: 13.29 ± 5.024) (Table 2).

**Table 2:** Results of the differences between sports in the AAQ test scores.

*N* = 24	Basketball (*n* = 9)		Table tennis (*n* = 8)		Handball (*n* = 7)		*p*
	Mean	SD	Mean	SD	Mean	SD	
Somatic anxiety	4.44	2.35	4.75	1.49	4.71	1.89	0.941
Cognitive anxiety	10.44	4.45	14.50	2.73	13.29	5.02	0.142
Self confidenece	16.78	4.15	16.50	3.11	17.43	1.98	0.859
Concentration	15.11	2.31	16.13	2.23	15.43	2.29	0.656

SD, standard deviation.

## 4 Discussion

Research on the effects of virtual reality (VR) is a modern and innovative field. Although VR has been used in sports research since the 1990s (36), it has recently gained significant attention again (54–56). The aim of our study was to assess athletes' psychological responses to a stress situation created in virtual reality (VR sports stress scenario). We hypothesized that the stress situation created in virtual reality would trigger similar stress responses in athletes as those experienced during competitions. Based on our results, we found no significant differences in any factor between VR and competitive matches. The results also indicated that the impact of VR on anxiety varied across different factors. While the stress-inducing effect of VR in terms of physical symptoms like trembling was relatively minimal, its influence on cognitive anxiety, particularly concerning performance fears, was notably stronger, reflected in the higher scores compared to those during the match. Thus, in this case, VR was able to induce higher cognitive anxiety than athletes typically experience during a competition. Based on this, we can conclude that a sports stress situation created in virtual reality can induce the same level of stress as competitive matches, indicating that VR could be a potentially valuable tool for improving athletes' stress management and as an important aid in sports psychological preparation. According to the stress inoculation theory, if a person is repeatedly exposed to stressors, it can reduce the intensity of their response to stress and help them adapt better to situations.

The goal of our research was to apply VR technology as effectively as possible. We created virtual environments for athletes that scientifically validated stress responses, such as the presence of opponents (48). Our aim was also for athletes to be able to influence the virtual reality, in contrast to other studies where this was not achieved (40). In their study, a goalkeeper observed a virtual environment where player took a penalty kick, and they were expected to move in the direction of the expected ball, but their movement did not influence the kicker's actions. In our study, the athlete was not simple a passive participant but was able to influence the course of the sports situation through their performance. The task was performed on a treadmill, and the faster the athlete ran, the faster they moved forward in virtual reality, thus influencing the events in the virtual environment. VR simulation programs are increasingly being integrated into sports psychology and performance-enhancing applications because they provide real-time interaction and feedback, which is crucial for the development of athletes. With VR simulation programs, athletes can practice not only their physical performance but also their mental and tactical aspects. This real-time interaction allows them to receive immediate feedback on their performance, which can contribute to faster development and performance optimization.

Although VR offers many benefits for sports psychological preparation, there are still many questions and limitations regarding the method's application. One of the most important questions is how accurately virtual environments can simulate psychological and physiological reactions of real competition situations. In VR environments, athletes face stressful situations, and the complex sensory effects experienced during real competitions—such as the presence of real opponents, audience reactions, physical exertion, and other sources of stress—are not always replicated. While VR simulations can influence the psychological responses of athletes, they may not fully replicate the dynamics of real events, especially in sports where physical interactions and tactical decisions play a key role. Therefore, these types of simulations are more likely to serve as complementary tools rather than completely replace real competition situations.

Another critical point is that technological and logistical limitations of VR may affect the widespread application of the method. Although VR simulations are very promising, they are often costly and require technical resources not available to every athlete. The development of the appropriate equipment and environments can be expensive and time-consuming, which could hinder the spread and everyday use of the method. Furthermore, as VR technology is continuously evolving, updates to the equipment and software, as well as the optimization of the user experience, may present ongoing challenges for researchers and coaches.

It is also essential to consider individual differences. While VR simulations may become a useful stress management tool for some athletes, others may not experience the same effect. For some athletes, virtual stress situations may be less effective as they may not be able to replicate the intensity of feelings and reactions experienced during real competitions. Therefore, future research should take into account athlete-specific differences and develop personalized VR programs so that virtual reality can become an optimal stress management and performance-enhancing tool for every athlete.

In conclusion, while the application of virtual reality in sports psychological preparation and stress management is highly promising, further development and refinement of the method are needed for it to become a more widely accessible and effective tool. With technological advancements and athlete-specific approaches, virtual reality may be able to contribute even more to improving athletes' performance and stress management abilities in the future.

### 4.1 Limitations

A limitation of this research is the small sample size and convenience sampling. In the future, it would be beneficial to repeat the study with randomized sampling and a larger sample size to obtain more accurate results and conclusions. Additionally, we would supplement our research by including both male and female athletes in the sample, allowing for the examination of gender differences as well. Furthermore, our goal is to broaden our research by increasing the number of participants and expanding the study across various sports. This approach will enable us to effectively measure differences between sports. Moreover, it can be argued that the use of a treadmill may not be an appropriate tool for every sport. Table tennis is a sport characterized by quick, precise movements and low-intensity dynamic actions, making the treadmill not necessarily the most optimal choice. For table tennis players, the use of a treadmill is less relevant as it does not closely align with the movements typical of the sport and does not promote the development of sport-specific skills.

Looking ahead, further research should explore how practicing VR sport stress scenarios might impact anxiety levels in high-pressure situations. We posit that repeated practice and task repetition, enabled by VR sports stress scenarios, can enhance learning processes, facilitating the adaptation necessary for development. Training under stress conditions builds resilience and prepares athletes for high-pressure real-world situations.

Developing various scenarios is crucial to avoid adaptation to just one situation and to ensure sport-specific preparation. The foundation of stress training lies in enabling athletes to adapt to diverse stress scenarios. Sport specificity is crucial because sports vary significantly in their demands. Practicing in a manner specific to one's sport can greatly enhance performance.

## 5 Conclusion

Our aim was to examine and assess the psychological responses elicited from athletes by the stress situation in virtual reality.Our results showed that the sports stress situation created in virtual reality elicits the same level of stress responses from the athletes as those experienced by the athletes in a competitive match, so virtual reality could be suitable for helping athletes manage stress. We believe that future measurements could have numerous practical benefits, even for coaches in selecting athletes who perform best under pressure situations. Coaches can provide real-time feedback and fine-tune athletes' stress management skills. In conclusion, we believe that VR-generated sport stress situations could effectively enhance athletes' stress training and integrate seamlessly into the process of sport psychological preparation, by enabling athletes to develop coping strategies that they can effectively apply in real sports environments. Furthermore we did not find a significant difference between those who work with a sports psychologist and those who do not, this may suggest that working with a sports psychologist does not immediately result in measurable differences in anxiety levels. However, this does not necessarily mean that the work of sports psychologists is ineffective; rather, it suggests that effectiveness may emerge over time, requiring longer-term studies. It also indicates that the impact may depend on other factors, such as the athlete's motivation, individual sensitivity, and the methods applied. Furthermore, the small sample size may have contributed to these results. There was also no significant difference in anxiety scores among athletes from the three sports, which may indicate that the type of sport is not a determining factor in anxiety levels. This is an important finding, as it suggests that similar approaches can be applied to manage anxiety across different sports. The effectiveness of the work of sports psychologists should not be judged solely based on these results. Further research is needed to consider long-term effects, individual differences, and the types and frequency of specific psychological interventions. At the same time, the results highlight that managing anxiety and achieving optimal performance is a complex process involving the interplay of multiple factors.

## Data Availability

The raw data supporting the conclusions of this article will be made available by the authors, without undue reservation.
